# Supplementary material for: An in-silico approach to predict and exploit synthetic lethality in cancer metabolism
Source: Nat Commun. 2017 Sep 6;8:459. doi: 10.1038/s41467-017-00555-y (PMC5587678; doi:10.1038/s41467-017-00555-y)
Supplement: Supplementary file 1 — Supplementary Information [file 41467_2017_555_MOESM1_ESM.pdf]

Description of Supplementary Files

File name: Supplementary Information
Description: Supplementary figures, supplementary tables, supplementary notes and supplementary references.

File name: Supplementary Data 1
Description: Results of calculations.

File name: Supplementary Software
Description: Matlab code used for the calculation of gMCSs.

File name: Peer review file

Supplementary Note 1. Illustration of our gMCSs methodology for predicting gene essentiality and conceptual comparison with existing approaches in the literature.

Our gMCSs approach is illustrated in Supplementary Figure 1. We first explain how gene expression data is integrated into our gMCS framework, and, second, the need to move from MCSs to gMCSs. Note here that MCSs, as defined in Klamt *et al.*[1], are minimal subsets of reactions (and not genes) whose simultaneous removal disrupts a particular metabolic task. Given the non-trivial nature of gene-protein-reaction (GPR) rules, we illustrate below that the mapping of MCSs at the gene level does not necessarily lead to gMCSs.

Supplementary Figure 1a shows an example metabolic network consisting of 9 reactions (r_2 is reversible). Here, for simplicity, we will suppose that each reaction is associated to a single gene and *vice versa*. As a consequence, in this case, computing MCSs will be equivalent to calculating gMCSs. We denote L the subset of reactions associated with lowly expressed genes. Similarly, $\overline{L}$ is the complement of L, which includes reactions associated to moderately/highly expressed genes or reactions lacking gene annotation.

The target reaction in this network is r_8. Assume that, as a result of previous research, the gene associated to r_2 is of interest and potentially related with r_8 in the context analyzed. Our objective is to compute MCSs containing r_2 which make reaction r_8 be inactive. As in Tobalina *et al.*[2], our approach is sufficiently flexible to directly search for MCSs involving a particular gene/reaction knockout of interest.

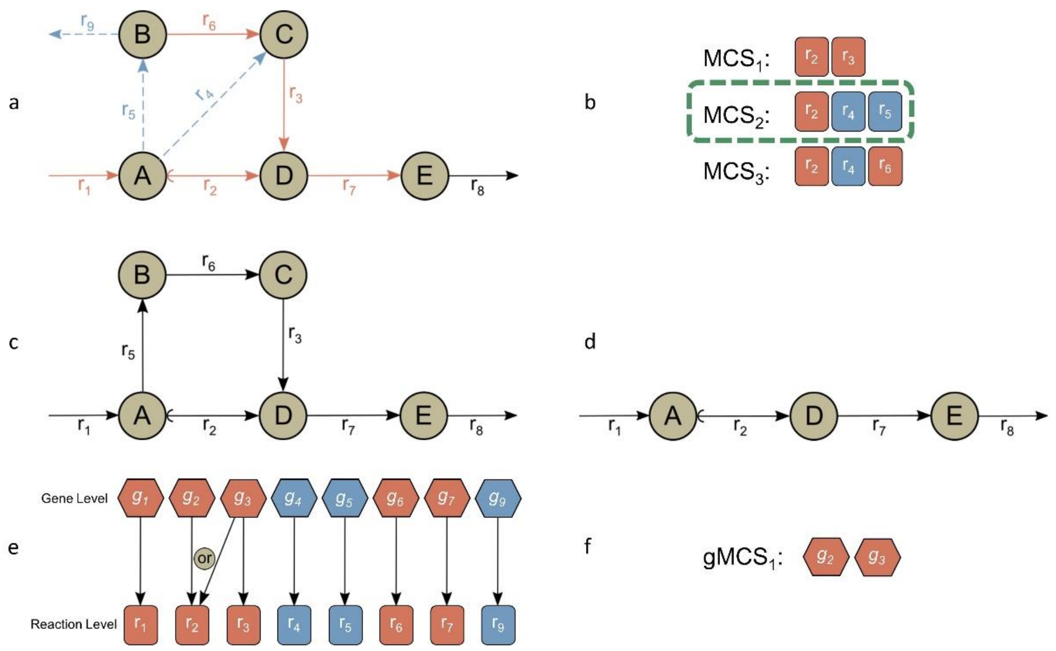

Supplementary Figure 1: Illustrative example of the effect of gene expression data in the computation of MCSs and the need for gMCSs. (a) Example metabolic network and reaction classification based on gene expression data. Blue dashed lines represent reactions associated with lowly expressed genes, L={4, 5, 9}, and red solid lines represent reactions associated with moderately/highly expressed genes or reactions lacking gene annotation, $\overline{L}$={1, 2, 3, 6, 7}. The target reaction is r_8; **(b)** Enumeration of MCSs involving r_2 based on reaction length, following Tobalina *et al.*[2]. Our approach, based on gene expression data, only determines MCS_2 (green dashed box), which explains the essentiality of r_2; **(c-d)** Contextualized networks when using iMAT[3] and GIMME[4] algorithms, respectively; **(e)** Non-trivial gene-protein-reaction (GPR) rules scenario for Supplementary Figure 1a; **(f)** Resulting gMCSs involving g_2 given the GPR rules in Supplementary Figure 1e.

There exist 3 MCSs which fulfill the aforementioned requirements: $MCS_1=\{r_2, r_3\}$; $MCS_2=\{r_2, r_4, r_5\}$; and $MCS_3=\{r_2, r_4, r_6\}$. Using the method presented in Tobalina *et al.*[2], these MCSs would be enumerated in the order they have been written above (Supplementary Fig. 1b). Notice that, in spite of being the shortest one, MCS_1 is formed by two reactions in $\overline{L}$ and, therefore, it is not able to explain the potential lethality of r_2. MCS_2, on the other hand, consists of one reaction in $\overline{L}$ and two reactions in L. Despite the fact that it contains more reactions than MCS_1, it is a much more interesting

synthetic lethal as it will only require blocking r_2 to prevent the activity of r_8, justifying the essential role of r_2 in this context.

With respect to Tobalina *et al.*[2], our approach directly calculates MCSs (if any exist) of this type, namely where all their reactions involved are in L, except for the one we are interested to target (r_2). This is particularly suitable for large metabolic networks, where full enumeration of MCSs is not computationally viable and the direct search of solutions of interest is required. Using this strategy, we can more efficiently evaluate the essentiality of a gene knockout for biomass production in a given context characterized by gene expression data.

In summary, when applied our methodology to this toy example, the second MCS involves r_2 ($\overline{L}$), r_4 (L) and r_5 (L), meaning that, as r_4 and r_5 have low activity according to gene expression data, r_2 plays an essential role. In other words, the single knockout of r_2 would render impossible any flux through the target reaction, namely, r_8. It is interesting to highlight that the computation of MCSs (or gMCSs) allows us, apart from predicting essentiality, to explain why a given reaction (or gene) is essential in a particular context. In this case, r_2 is essential for the activity of r_8 because r_4 and r_5 have low activity according gene expression data.

Reconstruction-based algorithms for gene essentiality analysis. The aim of the reconstruction methods is to identify a subset of reactions from the reference metabolic network that best fits to available expression data and satisfies steady-state condition, thermodynamic constraints and biomass production (in our case r_8). Once the reference network is contextualized using gene expression data, gene essentiality analysis is conducted, *i.e.* identification of single gene knockouts that disrupt biomass production. In particular, we will focus on two of the most common algorithms in the literature:

GIMME[4] and iMAT[3]. For illustration, we will evaluate the essentiality of r_2 in the resulting contextualized networks from these two approaches.

iMAT. In this method a particular reaction is removed from the reference metabolic network if, when it is blocked, its consistency with gene expression data is strictly higher than when it is forced to be active. To measure the consistency with gene expression data, iMAT gives the same weight to include a reaction in H (subset of highly expressed reactions) as to exclude a reaction in L.

For instance, if we delete r_4, the maximum consistency score with gene expression data would be 6 (agreement with r_1, r_2, r_3, r_4, r_6, r_7), while, if we activate r_4, this score would be 5 (agreement with r_1, r_2, r_3, r_6, r_7). In light of this, r_4 is excluded from the reconstruction. The same procedure is applied to each reaction. If both scores obtain the same result, the reaction is included in the reconstruction.

When applying iMAT to our toy example, we obtain the sub-network shown in Supplementary Figure 1c. The essentiality of r_2 is not predicted following the iMAT algorithm, since it has an escape pathway through r_3, r_5, r_6.

Note here that, in order to evaluate the performance of iMAT in the Results section of the main text, given its high computational demand, we had to introduce modifications with respect to the original version of the iMAT presented in Shlomi *et al.*[3] (see Supplementary Note 2).

GIMME. This approach first calculates an inconsistency score for each reaction in L, as a function of the difference between its expression level and the threshold which determines if a reaction is expressed or not. As reactions in L are not supposed to take part in the reconstruction, this algorithm includes all reactions in $\overline{L}$ and some reactions in L which minimize the sum of inconsistency scores. This minimization problem must satisfy steady-state condition, thermodynamic constraints and biomass production.

Supplementary Figure 1d shows the reconstructed network obtained following GIMME. This method does predict the essential role of r_2, that is, there are not alternative pathways to reach r_8 after knocking out r_2. Nevertheless, GIMME is not able to explain the reason why r_2 is essential, since when the reconstruction is conducted, r_4 and r_5 are removed from the solution network and, therefore, this information is lost.

Note here that, in order to evaluate the performance of GIMME in the Results section of the main text, we implemented the algorithm presented in Becker *et al.*[4]. As done with our gMCS approach, we used the Gene Expression Barcode 3.0 (ref. 5) to obtain the set lowly expressed genes, *L*.

Extending MCSs at the gene level (gMCSs). In contrast with existing methods for MCS computation[2,6], we extend the analysis to the gene level and determine gMCSs. It is important to emphasize that the subset of genes associated with the reactions involved in a particular MCS, determined using Gene-Protein-Reaction (GPR) rules, does not necessarily constitute a minimal knockout strategy. This is due to the fact that GPR rules are not always trivial (one-to-one association) and may involve complex relationships. In Recon2.v04 (ref. 7), for instance, this is the case for 88% of genes included. For illustration, assume that we are concerned in finding gMCSs involving g_2 for the toy metabolic network in Supplementary Figure 1a in a slightly more complex GPR rules scenario (Supplementary Fig. 1e). In this case, g_2 is only related to r_2, which can be catalyzed by one additional enzyme encoded by g_3; the rest of reactions are catalyzed by only one enzyme. In order to delete r_2 (the only potential effect over the network of knocking out g_2), we need to suppress g_2 and g_3 simultaneously and, when this is achieved, r_3 is indirectly deleted. As g_2 is necessarily coupled to g_3 to have any effect and they form a synthetic lethal, the knockout of g_4, g_5 or g_6 is not necessary any more to disrupt r_8.

Therefore, if we obtain the genes associated to MCS_1-MCS_3 using GPR rules, the only true minimal gene knockout solution would be $gMCS_1=\{g_2, g_3\}$ (Supplementary Fig. 1f). As g_3 is not included in the L set, our approach would lead to an 'infeasible' problem, *i.e.* with no solution. We conclude that g_2 is not essential for the activation of r_8 in this scenario, in disagreement to the solution previously achieved when the analysis restricted at the reaction level. With the use of gMCSs, our approach generalizes MCSs at the gene level, enabling the integration of complex GPR rules and overcoming issues considered above. Full details as to how the methodology presented in Tobalina *et al.*[2], was adapted to calculate gMCSs and incorporate gene expression data can be found in the Methods section of the main text.

Note here that for the study of *RRM1* in Multiple Myeloma (MM) conducted in the main text, we also calculated MCSs for *RRM1* (instead of gMCSs) for the four cell lines considered. When returned MCSs were mapped to the gene level, the obtained solutions in all cases were incorrect (6 out 6) (see Supplementary Data 1), *i.e.* certainly they were not gMCSs. This illustrates the importance of moving from MCSs to gMCSs, as we propose here.

Supplementary Note 2. Computational implementation of iMAT. The central optimization model proposed by iMAT for network reconstruction is the following mixed-integer linear programing (MILP):

$$\max\left(\sum_{i\in H}(y_i^+ + y_i^-) + \sum_{i\in L} y_i^+\right) \quad \text{(S1)}$$

Subject to:

$$\mathbf{S}\cdot\mathbf{v} = \mathbf{0} \quad \text{(S2)}$$

$$\mathbf{v_{min}} \leq \mathbf{v} \leq \mathbf{v_{max}} \quad \text{(S3)}$$

$$v_{biomass} \geq v_{biomass}^* \quad \text{(S4)}$$

$$v_i + y_i^+ \cdot \left(v_{min,\ i} - \varepsilon\right) \geq v_{min,\ i}, \quad \forall i \in H \quad \text{(S5)}$$

$$v_i + y_i^- \cdot \left(v_{max,\ i} + \varepsilon\right) \leq v_{max,\ i}, \quad \forall i \in H \quad \text{(S6)}$$

$$v_{min,\ i} \cdot (1 - y_i^+) \leq v_i \leq v_{max,\ i} \cdot (1 - y_i^+), \quad \forall i \in L \quad \text{(S7)}$$

$$\mathbf{v} \in R^n \quad \text{(S8)}$$

$$y_i^+, y_i^- \in \{0,1\}, \quad \forall i \in H \quad \text{(S9)}$$

$$y_i^+ \in \{0,1\}, \quad \forall i \in L \quad \text{(S10)}$$

, where H and L represents the subset of highly and lowly expressed reactions, respectively. For reactions in H, binary variables $y_i^+ = 1$ if $v_i \geq \varepsilon$, 0 otherwise; and $y_i^- = 1$ if $v_i \leq -\varepsilon$, 0 otherwise. For reactions in L, $y_i^+ = 1$ if $v_i = 0$, 0 otherwise. We fixed the same values of v_{min} and v_{max} used in our gMCS approach. Finally, $v_{biomass}^*$ represents the minimum flux required through the biomass reactions (here 0.001) and n denotes the number of reactions.

These optimization problem aims to strike a balance between the inclusion of H reactions and the exclusion of L reactions. Aware of the possible existence of alternative solutions, iMAT proposes an iterative solution scheme to assign a confidence score for the inclusion or exclusion of each reaction, namely comparing the objective value when i) $v_i = 0$ and ii) $v_i \neq 0$ ($v_i \geq \varepsilon \; or \; v_i \leq -\varepsilon$). With the current size of Recon2.v04 (ref. 7), this approach is prohibitive in terms of computation time, as we need to solve at least 2*n MILPs of similar complexity as the one shown above for each sample. To overcome this issue, we carried out the following implementation:

1) Solve the MILP shown above (equations (S1)-(S10)), extract the value of fluxes in the solution found (henceforth denoted as $\mathbf{u}$) and include non-zero fluxes in the output reconstruction. These active reactions must be part of the reconstruction since no better objective value can be found and, therefore, if they are knocked out, the objective value will be less or equal than the current one.

2) Evaluate whether other reactions without expression data available (set E), currently not part in the reconstruction, can be included. To that end, we force the fluxes in the same direction found as in the previous solution ($\mathbf{u}$), force to zero fluxes in H and L inactive in the previous solution and maximizes the number of reactions in E, which leads to the following MILP:

$$\max\left(\sum_{i \in E} z_i\right) \quad \text{(S11)}$$

Subject to:

$$\mathbf{S} \cdot \mathbf{v} = \mathbf{0} \quad \text{(S12)}$$

$$v_{min,\ i} \cdot z_i \leq v_i \leq v_{max,\ i} \cdot z_i\,, \quad i = 1, 2, \ldots, n \quad \text{(S13)}$$

$$v_{biomass} \geq v^{*}_{biomass} \quad \text{(S14)}$$

$$v_i \geq \varepsilon \text{ if } u_i \geq \varepsilon\,, \quad \forall i \in H, L \quad \text{(S15)}$$

$$v_i \leq -\varepsilon \text{ if } u_i \leq -\varepsilon\,, \quad \forall i \in H, L \quad \text{(S16)}$$

$$v_i = 0 \text{ if } u_i = 0\,, \quad \forall i \in H, L \quad \text{(S17)}$$

$$\mathbf{v} \in R^n \quad \text{(S18)}$$

$$z_i \in \{0, 1\}, \quad \forall i \in H, L \quad \text{(S19)}$$

With this second step, we keep the objective value found in Step 1 (given by reactions in H and L) and identify alternative pathways through reactions in E. This approach constitutes a computationally tractable approximation to iMAT. In terms of gene essentiality analysis, this approximation results in a best-case scenario, as we may have additional reactions in H and L that could be part of the reconstruction, which add new escape pathways and, therefore, would reduce the list of essential genes. In other words, with this implementation, a predicted non-essential gene is certainly non-essential in iMAT; however, a predicted essential gene could be non-essential in iMAT.

Supplementary Note 3. GPR rules for *RRM1*. As reported in the literature[8], *RRM1* constitutes the large regulatory subunit of the enzyme ribonucleotide reductase (RNR), which catalyzes the conversion of ribonucleoside diphosphates into deoxyribonucleoside diphosphates. *RRM1* binds to RRM2 or RRM2B to conduct metabolic activity. Recon2.v04 (ref. 7) correctly includes 4 cytosolic and 2 mitochondrial reactions for this conversion. However, their GPR rules are flawed. They are defined as (*RRM1* and *RRM2*) or (*RRM2B*), when it should read (*RRM1*) and (*RRM2* or *RRM2B*). The GPR rules for these reactions were corrected accordingly.

Recon2.v04 includes 4 additional cytosolic reactions associated with human RNR. These reactions convert ribonucleoside triphosphates into their corresponding deoxyribonucleoside triphosphates. These reactions, however, are not annotated in the literature to the human RNR[9]. In fact, these reactions are annotated in KEGG[10] to a different type of RNR, discovered in other organisms (http://www.genome.jp/dbget-bin/www_bget?ec:1.17.4.2). Given the complexity of biosynthesis and degradation pathways of deoxyribonucleotide triphophates, where new enzymes and reactions are discovered day by day[11], we used a conservative strategy and decided to keep these reactions with unknown GPR rules. Note that if these reactions were deleted from the reference metabolic network, *RRM1* becomes an essential gene for any type of cell, which is not in consonance with functional studies of *RRM1* silencing[12].

Supplementary Note 4. G matrix. As noted in the main text, we introduce the binary g x n matrix **G**, which defines for each row the set of blocked reactions arising from the knockout of a particular subset of genes in L. Genes associated with each row in **G** must be functionally interrelated and their simultaneous knockout is required to delete at least one of the reactions in the metabolic network.

For illustration, let us consider the toy example in Supplementary Figure 2, where we have four genes and six reactions. Blue color represents genes in L and reactions that become inactive when genes in L are knocked out (lowly expressed reactions). Red color shows genes in $\overline{L}$ and reactions potentially active even when genes in L are knocked out. For example, r_4 is not inactivated when genes in L are knocked out.

For matrix **G**, we only consider genes in L. For example, g_3 is not considered. Potential knockouts consist on all combinations without repetition of the genes in L. In our case, we have to analyze 7 different cases, namely, $\{g_1\}$, $\{g_2\}$, $\{g_4\}$, $\{g_1, g_2\}$, $\{g_1, g_4\}$, $\{g_2, g_4\}$ and $\{g_1, g_2, g_4\}$.

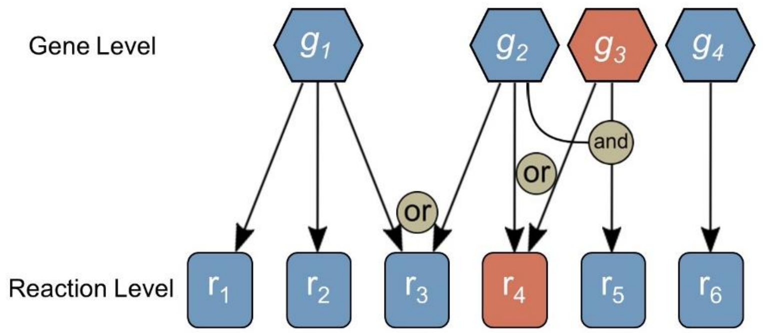

Supplementary Figure 2: Example GPR Rules and the calculation of its G Matrix. Enzyme encoded by g_1 catalyzes three different reactions: r_1, r_2 and r_3; r_3 is catalyzed by two different enzymes encoded by g_1 and g_2; r_4 is catalyzed by two different enzymes encoded by g_2 and g_3; r_5 is catalyzed by an enzymatic complex that comprises g_2 and g_3; r_6 is catalyzed by enzyme encoded by g_4.

The first step is about calculating which reactions become inactive when carrying out each of the 7 gene knockout combinations and introducing this information in the intermediate matrix **G'**. Regarding the GPR rules in Supplementary Figure 2, this will result in the following:

$$\mathbf{G}' = \begin{bmatrix} 1 & 1 & 0 & 0 & 0 & 0 \\ 0 & 0 & 0 & 0 & 1 & 0 \\ 0 & 0 & 0 & 0 & 0 & 1 \\ 1 & 1 & 1 & 0 & 1 & 0 \\ 1 & 1 & 0 & 0 & 0 & 1 \\ 0 & 0 & 0 & 0 & 1 & 1 \\ 1 & 1 & 0 & 0 & 1 & 1 \end{bmatrix} \begin{matrix} \{g_1\} \\ \{g_2\} \\ \{g_4\} \\ \{g_1, g_2\} \\ \{g_1, g_4\} \\ \{g_2, g_4\} \\ \{g_1, g_2, g_4\} \end{matrix} \qquad \text{(S20)}$$

Notice that, for example, r_3 can only be inactivated when knocking out g_1 and g_2 simultaneously, but either single deletion of these genes does not affect to the aforementioned reaction. However, just the contrary happens with $\{g_1, g_4\}$, $\{g_2, g_4\}$, $\{g_1, g_2, g_4\}$, meaning that the same set of reactions becomes inactive by combining at least two different rows in **G'**. For example, the combination of $\{g_1\}$ and $\{g_4\}$ inactivate the same set of reactions as $\{g_1, g_4\}$. As a consequence, the last three rows of **G'** are removed. The final **G** matrix is shown in equation (S21).

$$\mathbf{G} = \begin{bmatrix} 1 & 1 & 0 & 0 & 0 & 0 \\ 0 & 0 & 0 & 0 & 1 & 0 \\ 0 & 0 & 0 & 0 & 0 & 1 \\ 1 & 1 & 1 & 0 & 1 & 0 \end{bmatrix} \begin{matrix} \{g_1\} \\ \{g_2\} \\ \{g_4\} \\ \{g_1, g_2\} \end{matrix} \qquad \text{(S21)}$$

The naïve approach considered above which calculates all the possible combinations of genes in L is not computationally tractable. To overcome this issue, we have only calculated the combinations of genes in L related to each lowly expressed reaction. In the example discussed above, r_1, r_2, r_3, r_5 and r_6 are lowly expressed (colored blue), so the combinations of genes to study will be: g_1 related to r_1 and r_2; g_2 related to r_5; g_4 related to r_6; and $\{g_1, g_2\}$ related to r_3.

Note that all combinations containing g_3 have not been included because it is not lowly expressed. As a consequence, following this method we obtain the same **G** matrix in a more straightforward way.

The **G** matrix is used in our algorithm to define the potential list of reaction knockouts arising from the combination of genes in L (see equation (4) in the main paper).

Supplementary Figures.

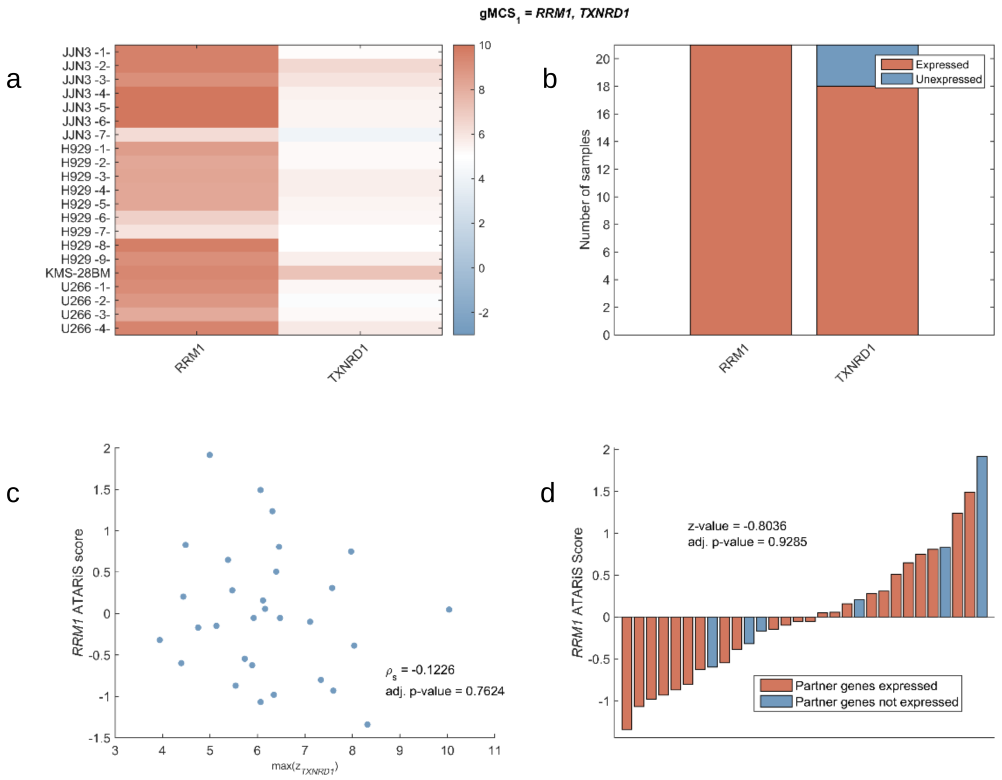

Supplementary Figure 3: $gMCS_1$ analysis. (a) Heatmap of Barcode z-scores[5] of *RRM1* and its partner genes (involved in $gMCS_1$) in different MM samples analyzed; **(b)** Number of MM samples where *RRM1* and its partner genes (involved in $gMCS_1$) are expressed/unexpressed, according to Barcode threshold of expression ($z \geq 5$). This gMCS explains the essentiality of *RRM1* in 3 samples; **(c)** Spearman´s correlation analyzing the dependence of *RRM1* ATARiS score on gene expression levels of partner genes in $gMCS_1$; **(d)** Bar plot of *RRM1* ATARiS score when partner genes are (or not) expressed in $gMCS_1$. To decide whether a gene is expressed, we used the standard threshold provided by the Gene Expression Barcode algorithm 3.0.

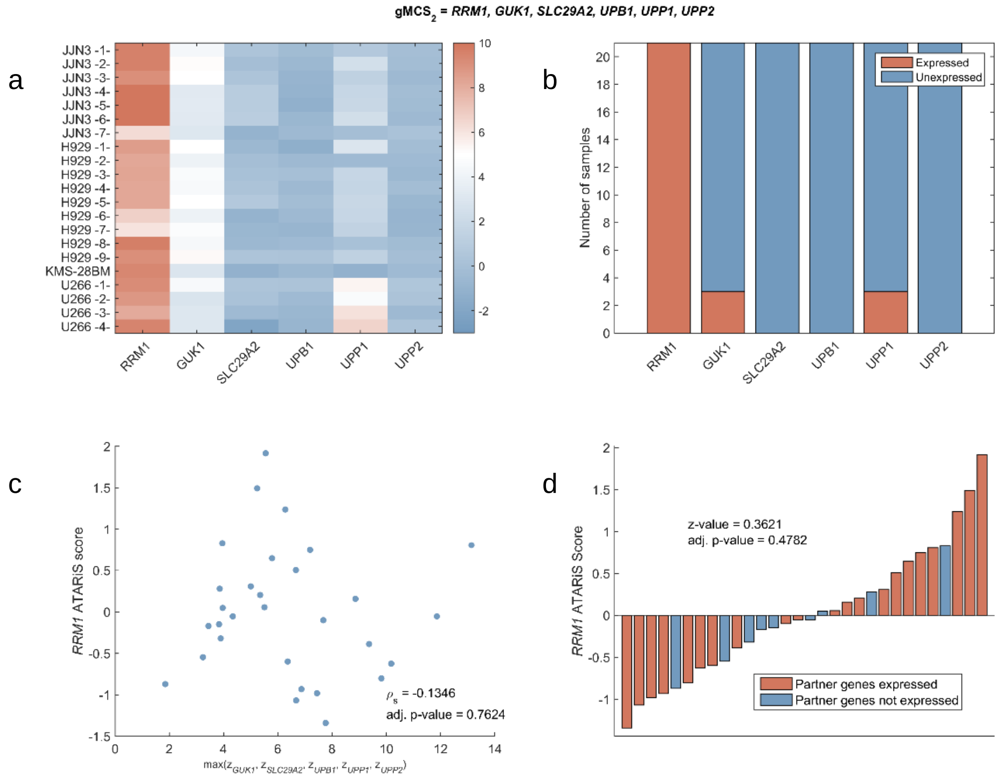

Supplementary Figure 4: gMCS$_2$ analysis. (a) Heatmap of Barcode z-scores[5] of *RRM1* and its partner genes (involved in gMCS$_2$) in different MM samples analyzed; **(b)** Number of MM samples where *RRM1* and its partner genes (involved in gMCS$_2$) are expressed/unexpressed, according to Barcode threshold of expression ($z \geq 5$). This gMCS explains the essentiality of *RRM1* in 15 samples; **(c)** Spearman´s correlation analyzing the dependence of *RRM1* ATARiS score on gene expression levels of partner genes in gMCS$_2$; **(d)** Bar plot of *RRM1* ATARiS score when partner genes are (or not) expressed in gMCS$_2$. To decide whether a gene is expressed, we used the standard threshold provided by the Gene Expression Barcode algorithm 3.0.

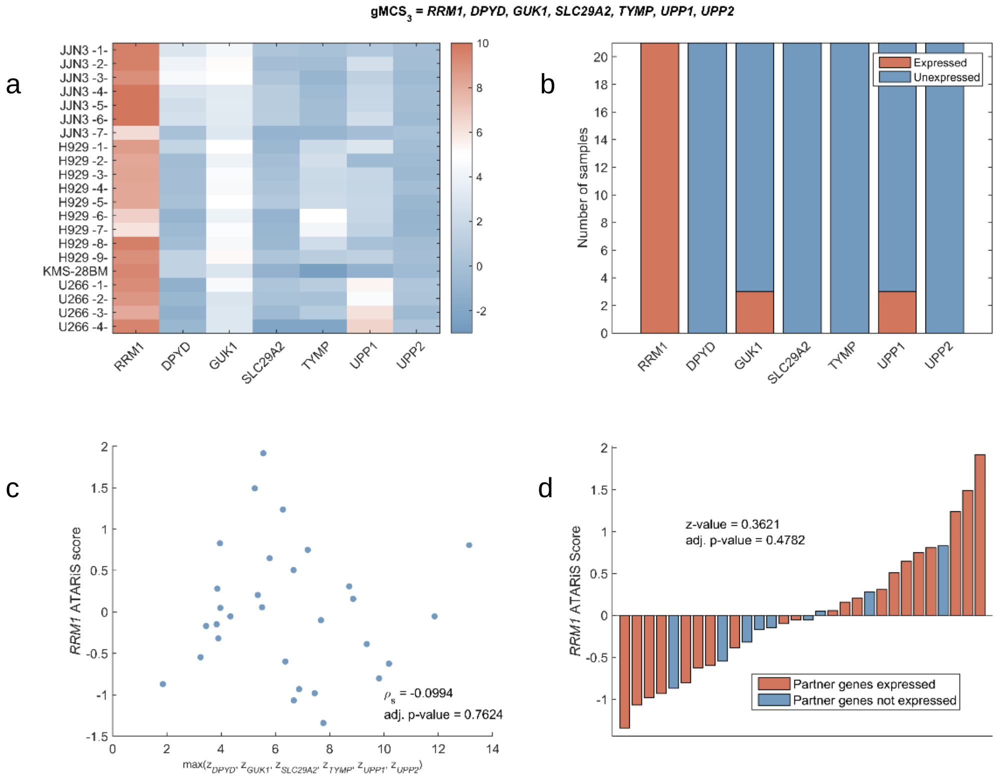

Supplementary Figure 5: gMCS$_3$ analysis. (a) Heatmap of Barcode z-scores[5] of *RRM1* and its partner genes (involved in gMCS$_3$) in different MM samples analyzed; **(b)** Number of MM samples where *RRM1* and its partner genes (involved in gMCS$_3$) are expressed/unexpressed, according to Barcode threshold of expression ($z \geq 5$). This gMCS explains the essentiality of *RRM1* in 15 samples; **(c)** Spearman´s correlation analyzing the dependence of *RRM1* ATARiS score on gene expression levels of partner genes in gMCS$_3$; **(d)** Bar plot of *RRM1* ATARiS score when partner genes are (or not) expressed in gMCS$_3$. To decide whether a gene is expressed, we used the standard threshold provided by the Gene Expression Barcode algorithm 3.0.

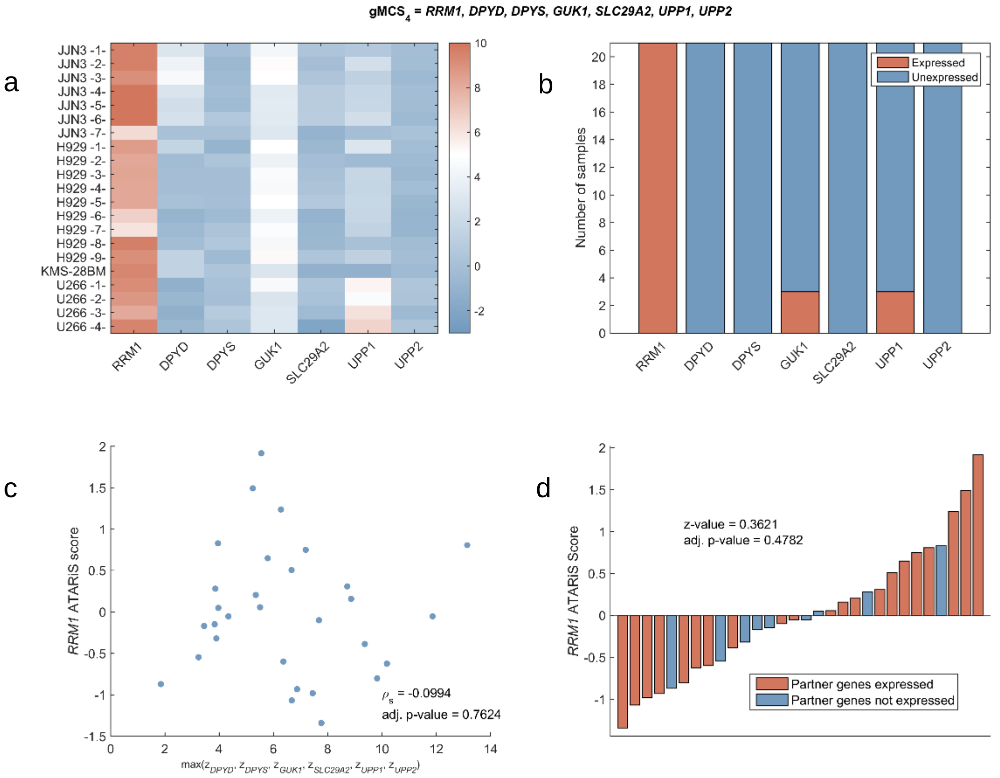

Supplementary Figure 6: gMCS$_4$ analysis. (a) Heatmap of Barcode z-scores[5] of *RRM1* and its partner genes (involved in gMCS$_4$) in different MM samples analyzed; **(b)** Number of MM samples where *RRM1* and its partner genes (involved in gMCS$_4$) are expressed/unexpressed, according to Barcode threshold of expression ($z \geq 5$). This gMCS explains the essentiality of *RRM1* in 15 samples; **(c)** Spearman´s correlation analyzing the dependence of *RRM1* ATARiS score on gene expression levels of partner genes in gMCS$_4$; **(d)** Bar plot of *RRM1* ATARiS score when partner genes are (or not) expressed in gMCS$_4$. To decide whether a gene is expressed, we used the standard threshold provided by the Gene Expression Barcode algorithm 3.0.

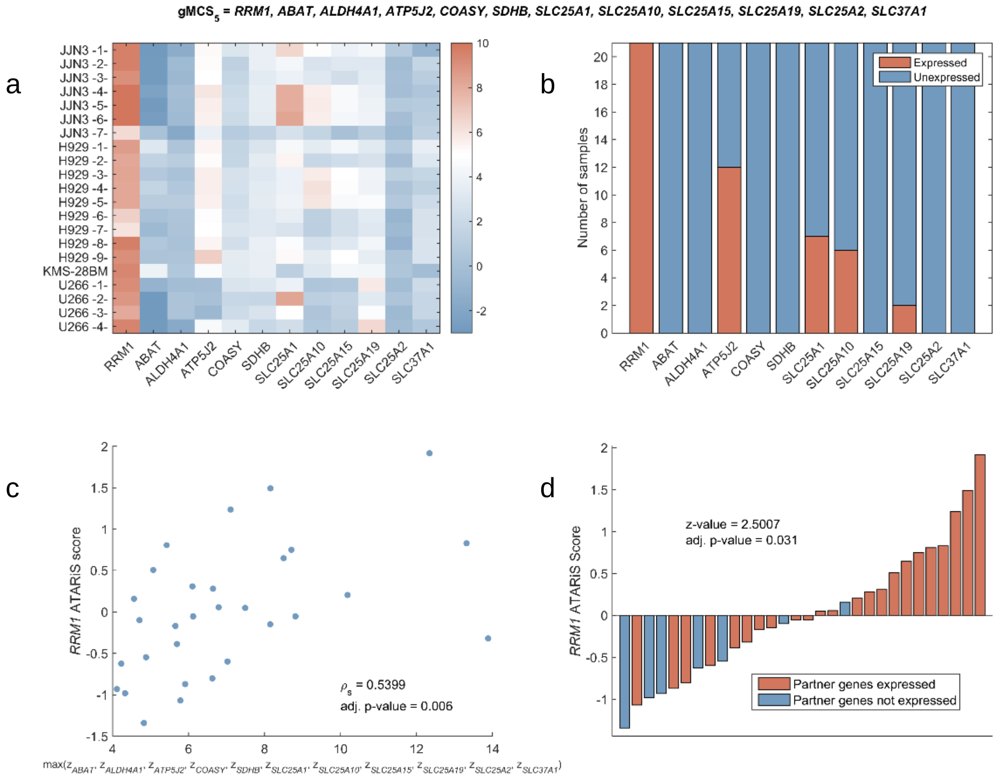

Supplementary Figure 7: gMCS$_5$ analysis. (a) Heatmap of Barcode z-scores[5] of *RRM1* and its partner genes (involved in gMCS$_5$) in different MM samples analyzed; **(b)** Number of MM samples where *RRM1* and its partner genes (involved in gMCS$_5$) are expressed/unexpressed, according to Barcode threshold of expression ($z \geq 5$). This gMCS explains the essentiality of *RRM1* in 6 samples; **(c)** Spearman´s correlation analyzing the dependence of *RRM1* ATARiS score on gene expression levels of partner genes in gMCS$_5$; **(d)** Bar plot of *RRM1* ATARiS score when partner genes are (or not) expressed in gMCS$_5$. To decide whether a gene is expressed, we used the standard threshold provided by the Gene Expression Barcode algorithm 3.0.

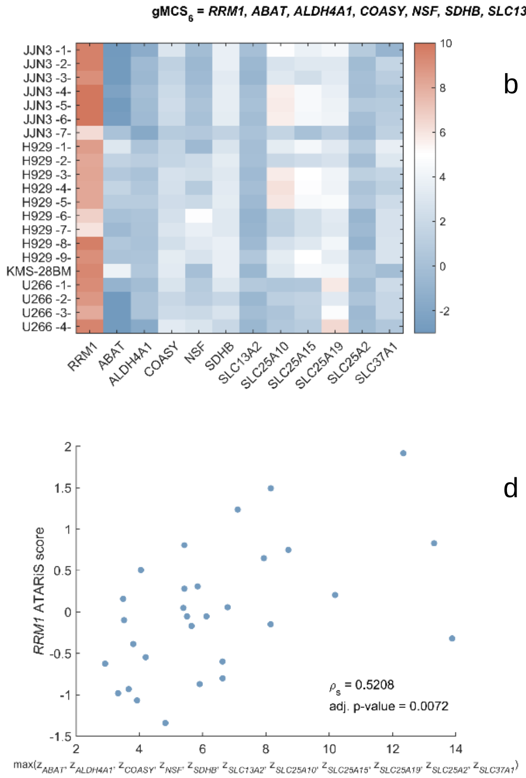

Supplementary Figure 8: gMCS$_6$ analysis. (a) Heatmap of Barcode z-scores[5] of *RRM1* and its partner genes (involved in gMCS$_6$) in different MM samples analyzed; **(b)** Number of MM samples where *RRM1* and its partner genes (involved in gMCS$_6$) are expressed/unexpressed, according to Barcode threshold of expression ($z \geq 5$). This gMCS explains the essentiality of *RRM1* in 13 samples; **(c)** Spearman´s correlation analyzing the dependence of *RRM1* ATARiS score on gene expression levels of partner genes in gMCS$_6$; **(d)** Bar plot of *RRM1* ATARiS score when partner genes are (or not) expressed in gMCS$_6$. To decide whether a gene is expressed, we used the standard threshold provided by the Gene Expression Barcode algorithm 3.0.

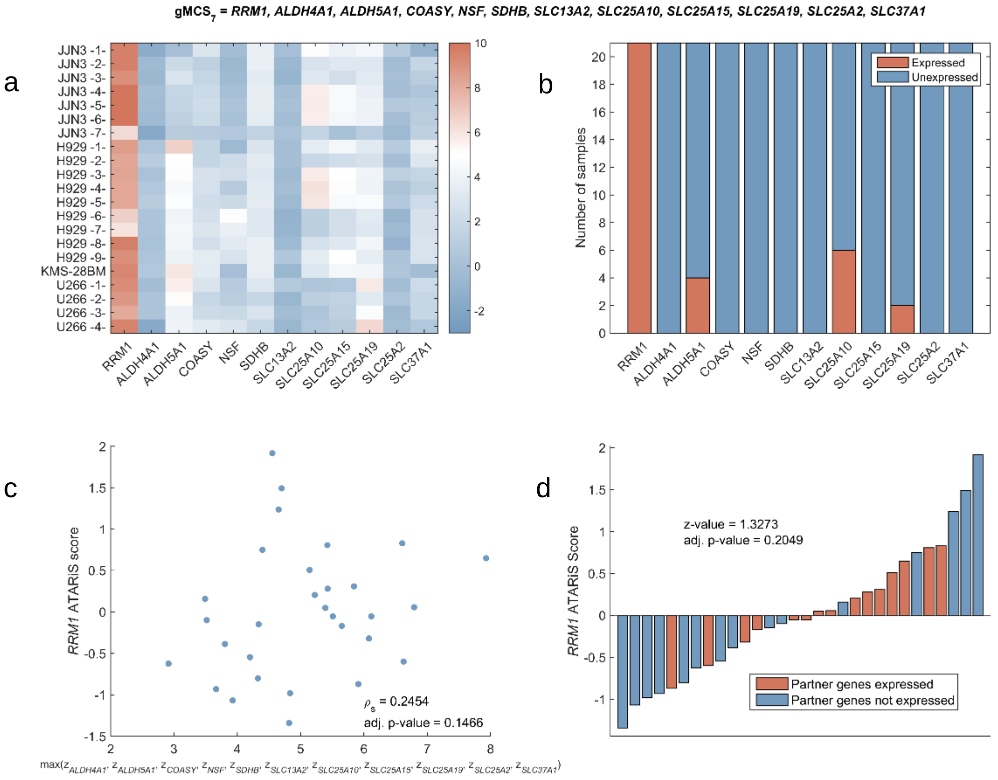

Supplementary Figure 9: gMCS$_7$ analysis. (a) Heatmap of Barcode z-scores[5] of *RRM1* and its partner genes (involved in gMCS$_7$) in different MM samples analyzed; **(b)** Number of MM samples where *RRM1* and its partner genes (involved in gMCS$_7$) are expressed/unexpressed, according to Barcode threshold of expression ($z \geq 5$). This gMCS explains the essentiality of *RRM1* in 10 samples; **(c)** Spearman´s correlation analyzing the dependence of *RRM1* ATARiS score on gene expression levels of partner genes in gMCS$_7$; **(d)** Bar plot of *RRM1* ATARiS score when partner genes are (or not) expressed in gMCS$_7$. To decide whether a gene is expressed, we used the standard threshold provided by the Gene Expression Barcode algorithm 3.0.

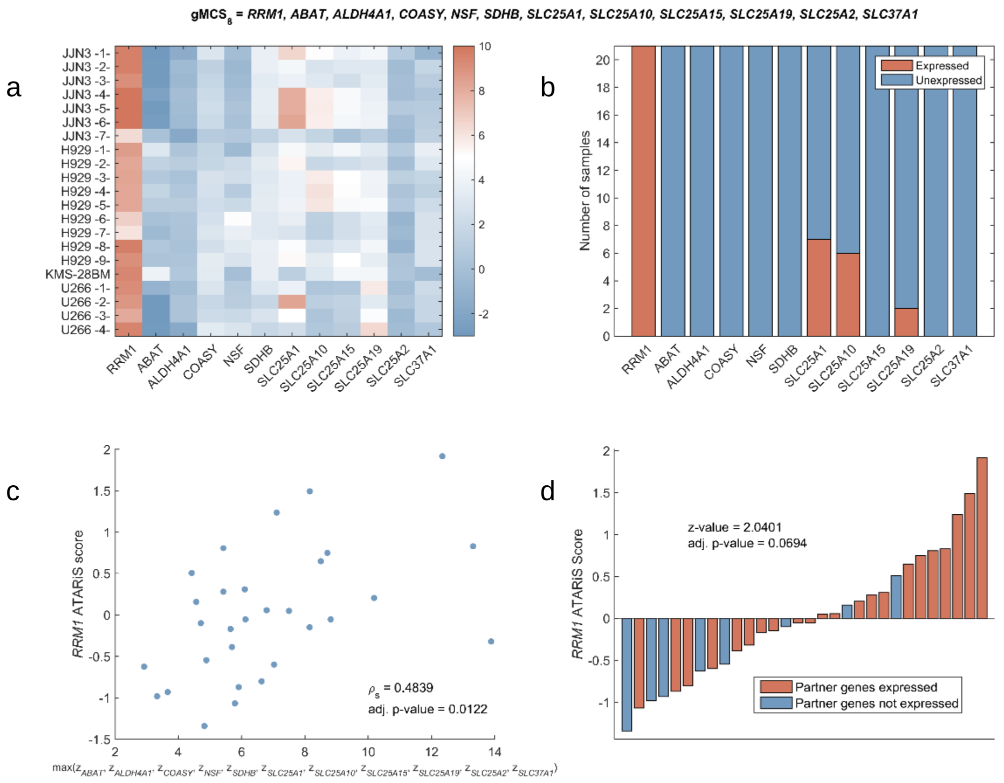

Supplementary Figure 10: gMCS$_8$ analysis. (a) Heatmap of Barcode z-scores[5] of *RRM1* and its partner genes (involved in gMCS$_8$) in different MM samples analyzed; **(b)** Number of MM samples where *RRM1* and its partner genes (involved in gMCS$_8$) are expressed/unexpressed, according to Barcode threshold of expression ($z \geq 5$). This gMCS explains the essentiality of *RRM1* in 9 samples; **(c)** Spearman´s correlation analyzing the dependence of *RRM1* ATARiS score on gene expression levels of partner genes in gMCS$_8$; **(d)** Bar plot of *RRM1* ATARiS score when partner genes are (or not) expressed in gMCS$_8$. To decide whether a gene is expressed, we used the standard threshold provided by the Gene Expression Barcode algorithm 3.0.

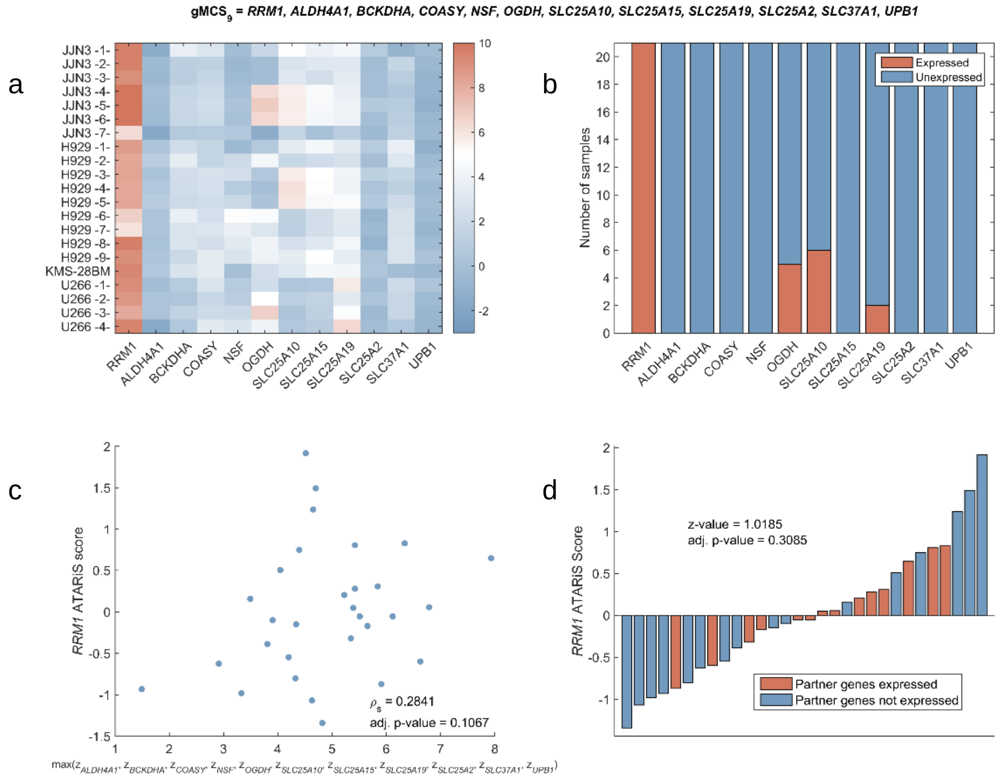

Supplementary Figure 11: gMCS$_9$ analysis. **(a)** Heatmap of Barcode z-scores[5] of *RRM1* and its partner genes (involved in gMCS$_9$) in different MM samples analyzed; **(b)** Number of MM samples where *RRM1* and its partner genes (involved in gMCS$_9$) are expressed/unexpressed, according to Barcode threshold of expression ($z \geq 5$). This gMCS explains the essentiality of *RRM1* in 11 samples; **(c)** Spearman´s correlation analyzing the dependence of *RRM1* ATARiS score on gene expression levels of partner genes in gMCS$_9$; **(d)** Bar plot of *RRM1* ATARiS score when partner genes are (or not) expressed in gMCS$_9$. To decide whether a gene is expressed, we used the standard threshold provided by the Gene Expression Barcode algorithm 3.0.

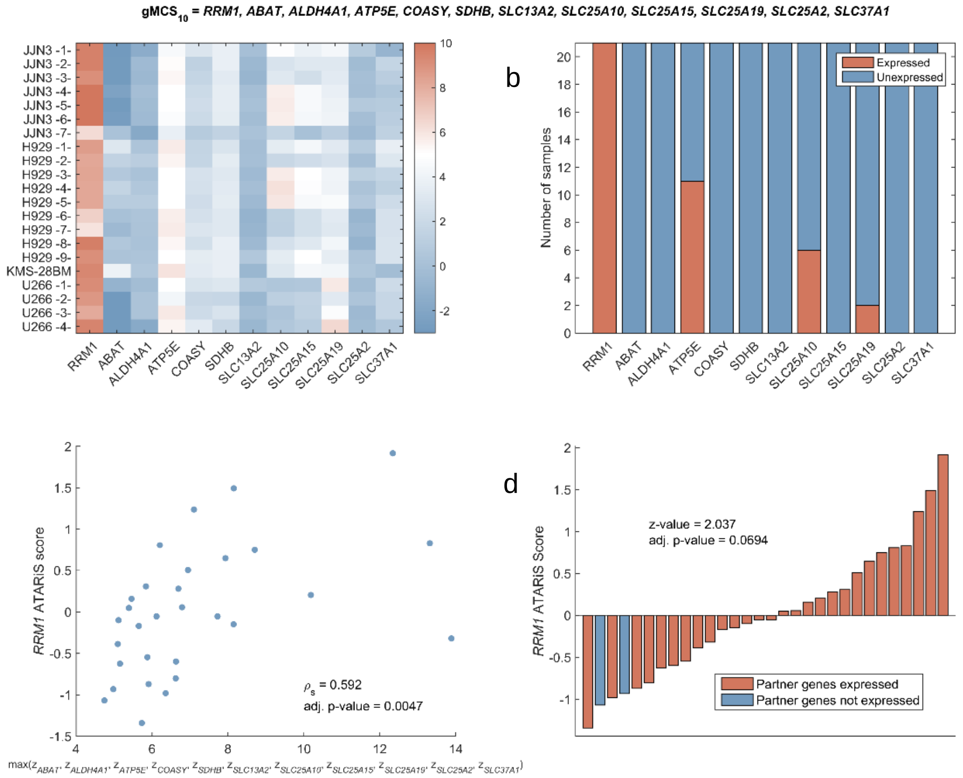

Supplementary Figure 12: gMCS$_{10}$ analysis. (a) Heatmap of Barcode z-scores[5] of *RRM1* and its partner genes (involved in gMCS$_{10}$) in different MM samples analyzed; **(b)** Number of MM samples where *RRM1* and its partner genes (involved in gMCS$_{10}$) are expressed/unexpressed, according to Barcode threshold of expression ($z \geq 5$). This gMCS explains the essentiality of *RRM1* in 3 samples; **(c)** Spearman´s correlation analyzing the dependence of *RRM1* ATARiS score on gene expression levels of partner genes in gMCS$_{10}$; **(d)** Bar plot of *RRM1* ATARiS score when partner genes are (or not) expressed in gMCS$_{10}$. To decide whether a gene is expressed, we used the standard threshold provided by the Gene Expression Barcode algorithm 3.0.

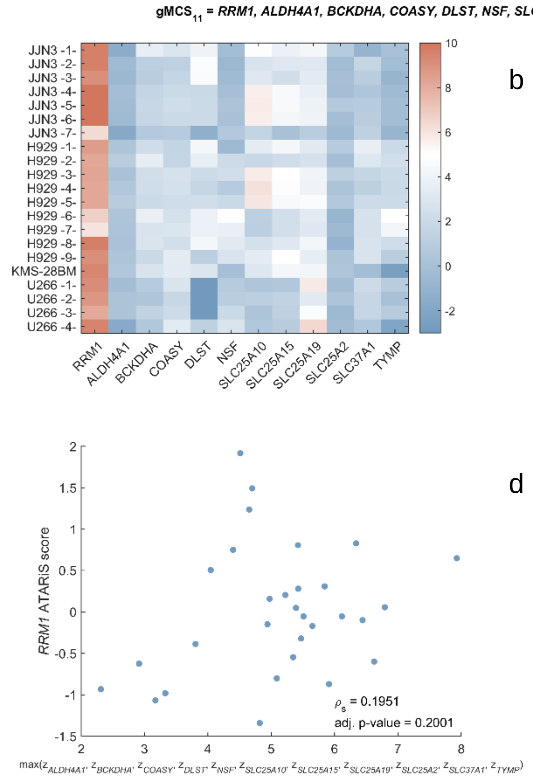

Supplementary Figure 13: gMCS$_{11}$ analysis. (a) Heatmap of Barcode z-scores[5] of *RRM1* and its partner genes (involved in gMCS$_{11}$) in different MM samples analyzed; **(b)** Number of MM samples where *RRM1* and its partner genes (involved in gMCS$_{11}$) are expressed/unexpressed, according to Barcode threshold of expression ($z \geq 5$). This gMCS explains the essentiality of *RRM1* in 13 samples; **(c)** Spearman´s correlation analyzing the dependence of *RRM1* ATARiS score on gene expression levels of partner genes in gMCS$_{11}$; **(d)** Bar plot of *RRM1* ATARiS score when partner genes are (or not) expressed in gMCS$_{11}$. To decide whether a gene is expressed, we used the standard threshold provided by the Gene Expression Barcode algorithm 3.0.

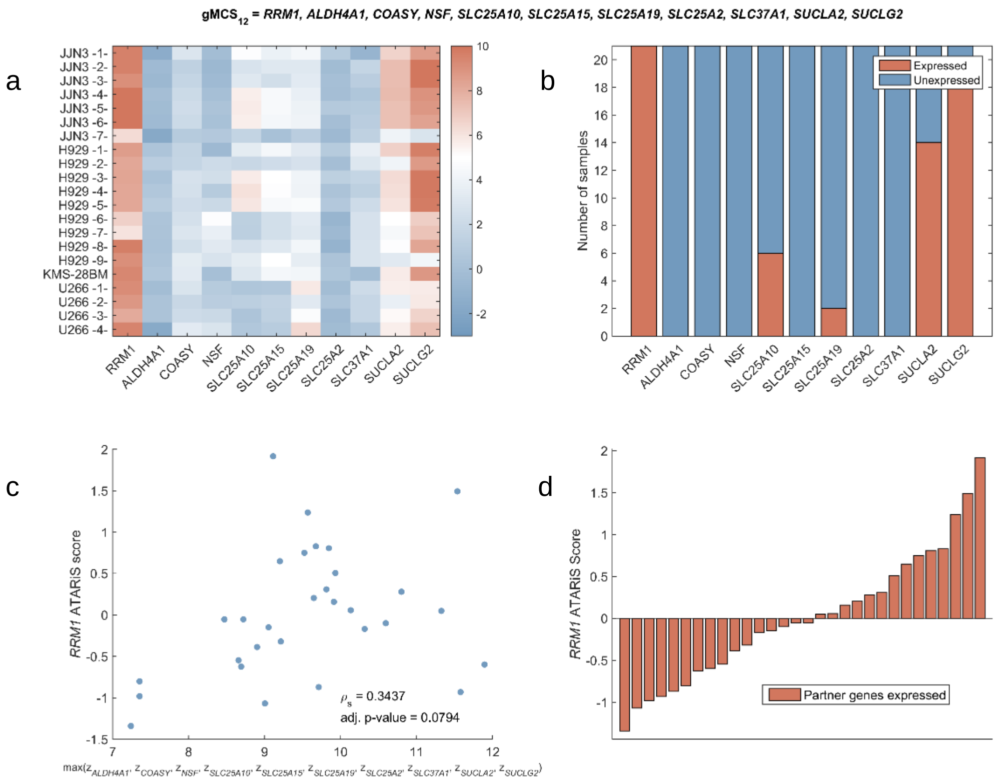

Supplementary Figure 14: gMCS$_{12}$ analysis. (a) Heatmap of Barcode z-scores[5] of *RRM1* and its partner genes (involved in gMCS$_{12}$) in different MM samples analyzed; **(b)** Number of MM samples where *RRM1* and its partner genes (involved in gMCS$_{12}$) are expressed/unexpressed, according to Barcode threshold of expression ($z \geq 5$). This gMCS explains the essentiality of *RRM1* in 2 samples; **(c)** Spearman´s correlation analyzing the dependence of *RRM1* ATARiS score on gene expression levels of partner genes in gMCS$_{12}$; **(d)** Bar plot of *RRM1* ATARiS score when partner genes are (or not) expressed in gMCS$_{12}$. To decide whether a gene is expressed, we used the standard threshold provided by the Gene Expression Barcode algorithm 3.0.

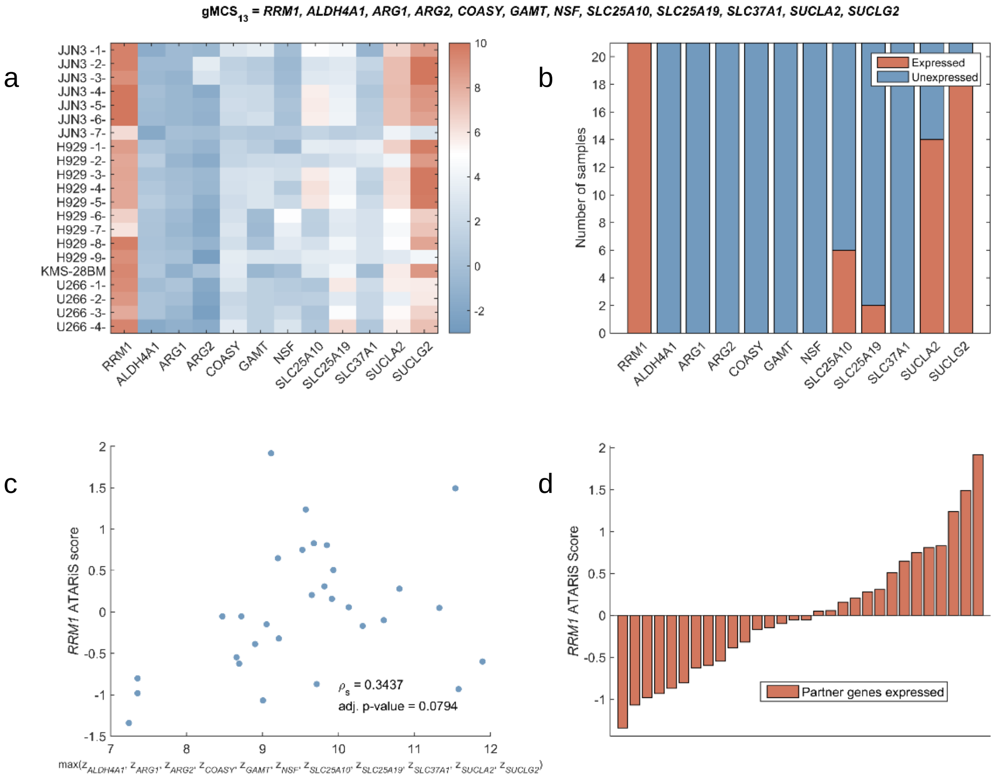

Supplementary Figure 15: gMCS$_{13}$ analysis. (a) Heatmap of Barcode z-scores[5] of *RRM1* and its partner genes (involved in gMCS$_{13}$) in different MM samples analyzed; **(b)** Number of MM samples where *RRM1* and its partner genes (involved in gMCS$_{13}$) are expressed/unexpressed, according to Barcode threshold of expression ($z \geq 5$). This gMCS explains the essentiality of *RRM1* in 2 samples; **(c)** Spearman´s correlation analyzing the dependence of *RRM1* ATARiS score on gene expression levels of partner genes in gMCS$_{13}$; **(d)** Bar plot of *RRM1* ATARiS score when partner genes are (or not) expressed in gMCS$_{13}$. To decide whether a gene is expressed, we used the standard threshold provided by the Gene Expression Barcode algorithm 3.0.

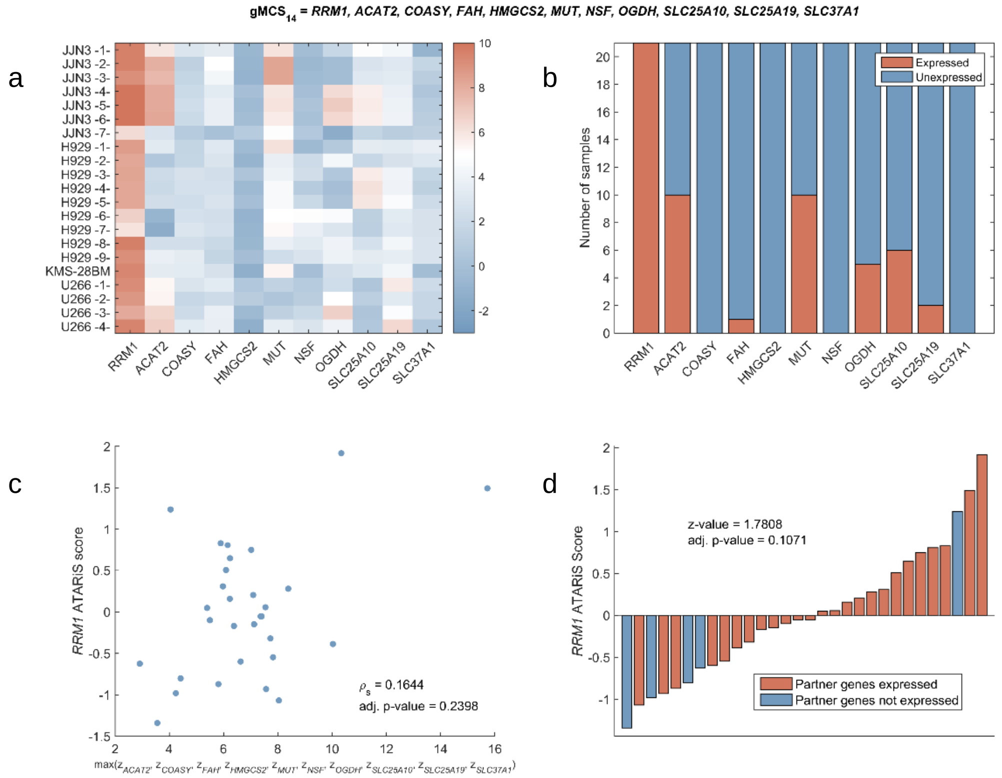

Supplementary Figure 16: gMCS$_{14}$ analysis. (a) Heatmap of Barcode z-scores[5] of *RRM1* and its partner genes (involved in gMCS$_{14}$) in different MM samples analyzed; **(b)** Number of MM samples where *RRM1* and its partner genes (involved in gMCS$_{14}$) are expressed/unexpressed, according to Barcode threshold of expression ($z \geq 5$). This gMCS explains the essentiality of *RRM1* in 4 samples; **(c)** Spearman´s correlation analyzing the dependence of *RRM1* ATARiS score on gene expression levels of partner genes in gMCS$_{14}$; **(d)** Bar plot of *RRM1* ATARiS score when partner genes are (or not) expressed in gMCS$_{14}$. To decide whether a gene is expressed, we used the standard threshold provided by the Gene Expression Barcode algorithm 3.0.

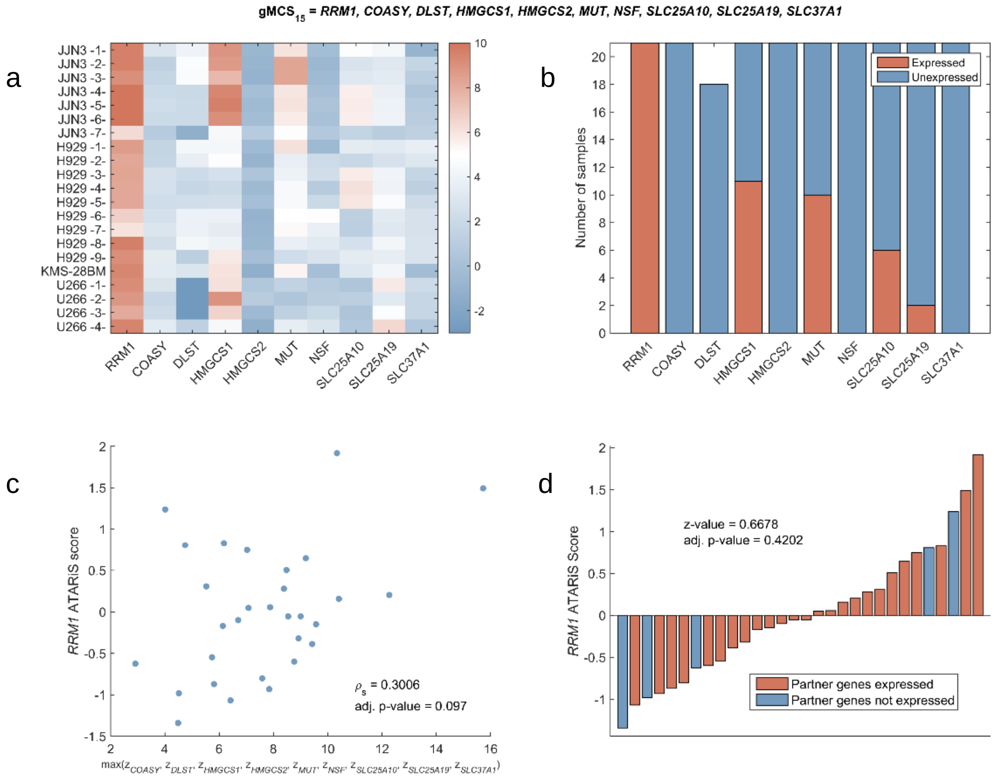

Supplementary Figure 17: gMCS$_{15}$ analysis. (a) Heatmap of Barcode z-scores[5] of *RRM1* and its partner genes (involved in gMCS$_{15}$) in different MM samples analyzed; **(b)** Number of MM samples where *RRM1* and its partner genes (involved in gMCS$_{15}$) are expressed/unexpressed, according to Barcode threshold of expression ($z \geq 5$). This gMCS explains the essentiality of *RRM1* in 3 samples; **(c)** Spearman´s correlation analyzing the dependence of *RRM1* ATARiS score on gene expression levels of partner genes in gMCS$_{15}$; **(d)** Bar plot of *RRM1* ATARiS score when partner genes are (or not) expressed in gMCS$_{15}$. To decide whether a gene is expressed, we used the standard threshold provided by the Gene Expression Barcode algorithm 3.0.

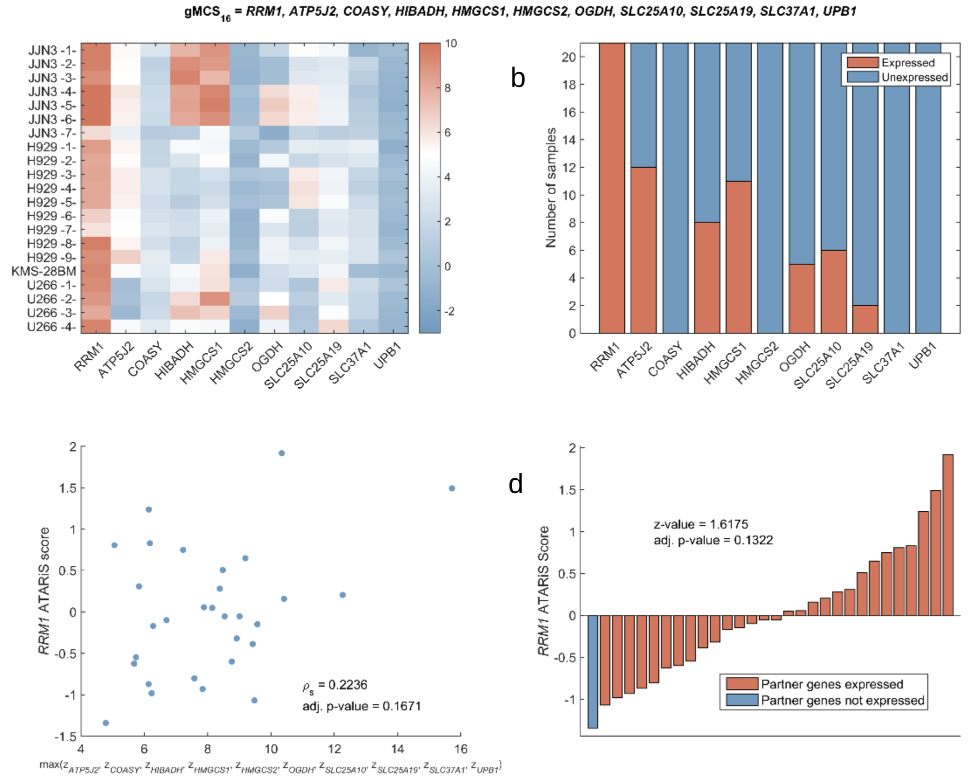

Supplementary Figure 18: gMCS$_{16}$ analysis. (a) Heatmap of Barcode z-scores[5] of *RRM1* and its partner genes (involved in gMCS$_{16}$) in different MM samples analyzed; **(b)** Number of MM samples where *RRM1* and its partner genes (involved in gMCS$_{16}$) are expressed/unexpressed, according to Barcode threshold of expression ($z \geq 5$). This gMCS explains the essentiality of *RRM1* in 3 samples; **(c)** Spearman´s correlation analyzing the dependence of *RRM1* ATARiS score on gene expression levels of partner genes in gMCS$_{16}$; **(d)** Bar plot of *RRM1* ATARiS score when partner genes are (or not) expressed in gMCS$_{16}$. To decide whether a gene is expressed, we used the standard threshold provided by the Gene Expression Barcode algorithm 3.0.

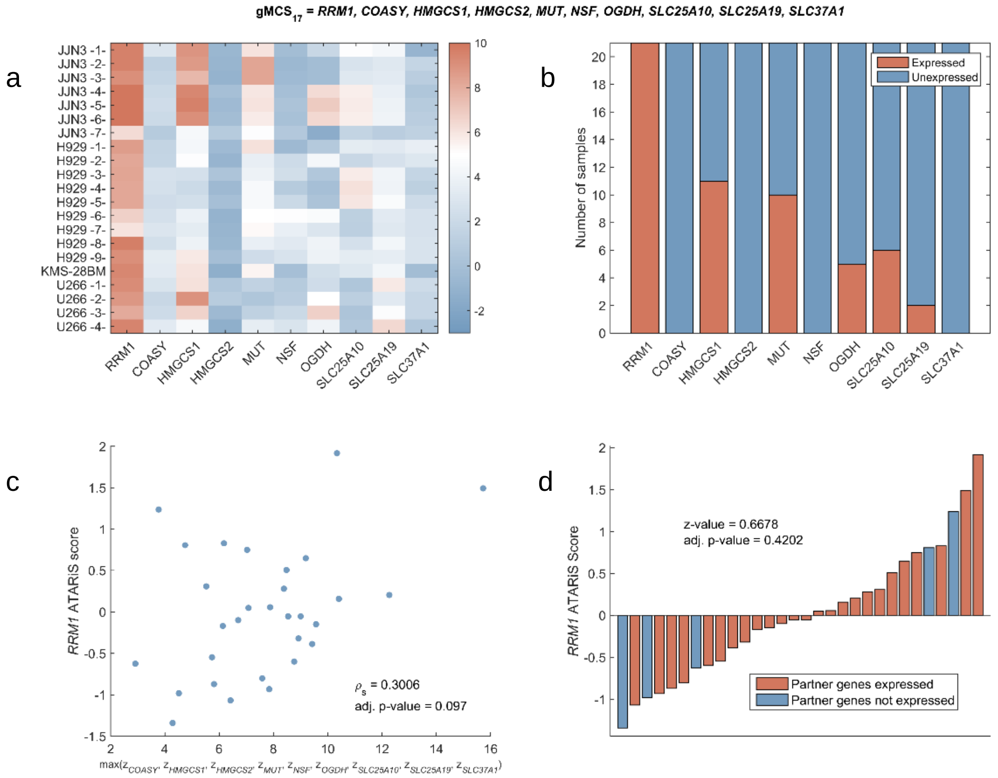

Supplementary Figure 19: $gMCS_{17}$ analysis. (a) Heatmap of Barcode z-scores[5] of *RRM1* and its partner genes (involved in $gMCS_{17}$) in different MM samples analyzed; **(b)** Number of MM samples where *RRM1* and its partner genes (involved in $gMCS_{17}$) are expressed/unexpressed, according to Barcode threshold of expression ($z \geq 5$). This gMCS explains the essentiality of *RRM1* in 3 samples; **(c)** Spearman´s correlation analyzing the dependence of *RRM1* ATARiS score on gene expression levels of partner genes in $gMCS_{17}$; **(d)** Bar plot of *RRM1* ATARiS score when partner genes are (or not) expressed in $gMCS_{17}$. To decide whether a gene is expressed, we used the standard threshold provided by the Gene Expression Barcode algorithm 3.0.

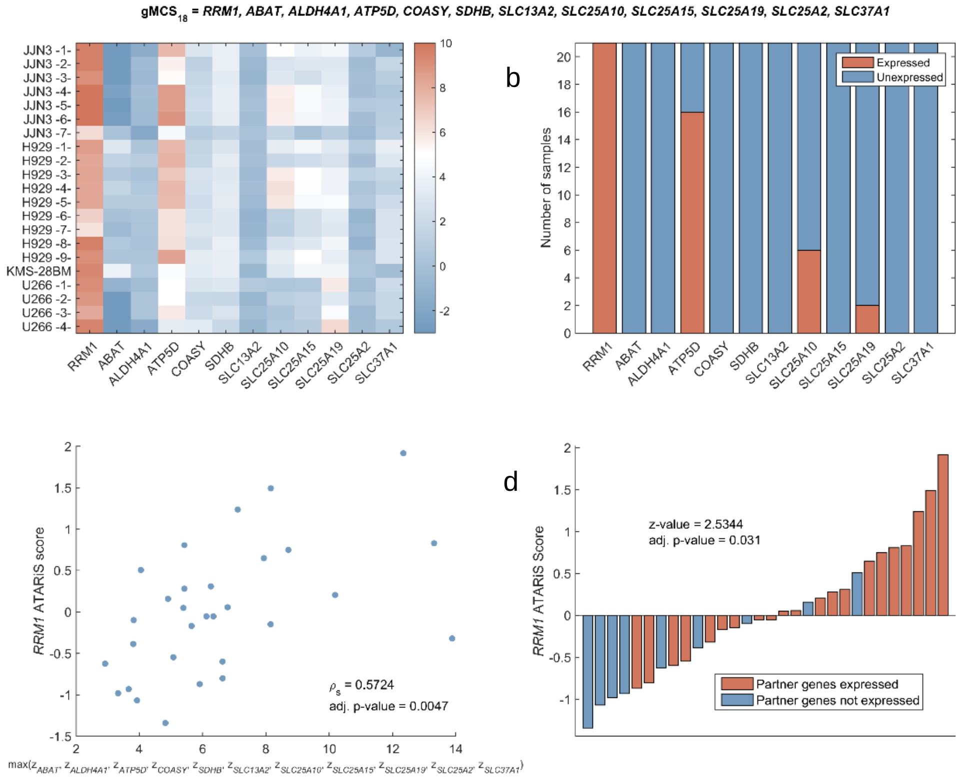

Supplementary Figure 20: gMCS$_{18}$ analysis. (a) Heatmap of Barcode z-scores[5] of *RRM1* and its partner genes (involved in gMCS$_{18}$) in different MM samples analyzed; **(b)** Number of MM samples where *RRM1* and its partner genes (involved in gMCS$_{18}$) are expressed/unexpressed, according to Barcode threshold of expression ($z \geq 5$). This gMCS explains the essentiality of *RRM1* in 3 samples; **(c)** Spearman´s correlation analyzing the dependence of *RRM1* ATARiS score on gene expression levels of partner genes in gMCS$_{18}$; **(d)** Bar plot of *RRM1* ATARiS score when partner genes are (or not) expressed in gMCS$_{18}$. To decide whether a gene is expressed, we used the standard threshold provided by the Gene Expression Barcode algorithm 3.0.

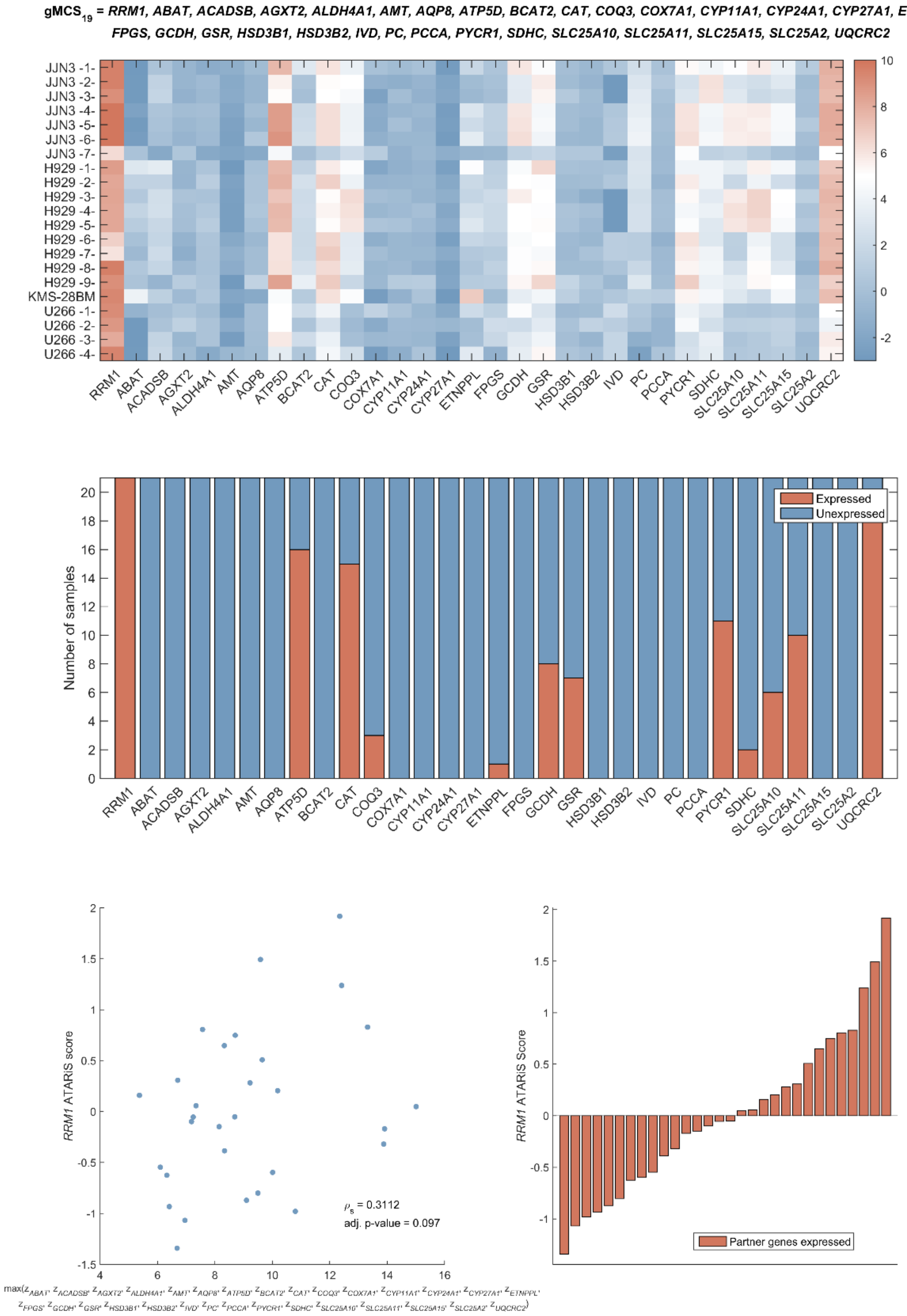

Supplementary Figure 21: gMCS$_{19}$ analysis. (a) Heatmap of Barcode z-scores[5] of *RRM1* and its partner genes (involved in gMCS$_{19}$) in different MM samples analyzed; **(b)** Number of MM samples where *RRM1* and its partner genes (involved in gMCS$_{19}$) are expressed/unexpressed, according to Barcode threshold of expression ($z \geq 5$). This gMCS explains the essentiality of *RRM1* in 3 samples; **(c)** Spearman´s correlation analyzing the dependence of *RRM1* ATARiS score on gene expression levels of partner genes in gMCS$_{19}$; **(d)** Bar plot of *RRM1* ATARiS score when partner genes are (or not) expressed in gMCS$_{19}$. To decide whether a gene is expressed, we used the standard threshold provided by the Gene Expression Barcode algorithm 3.0.

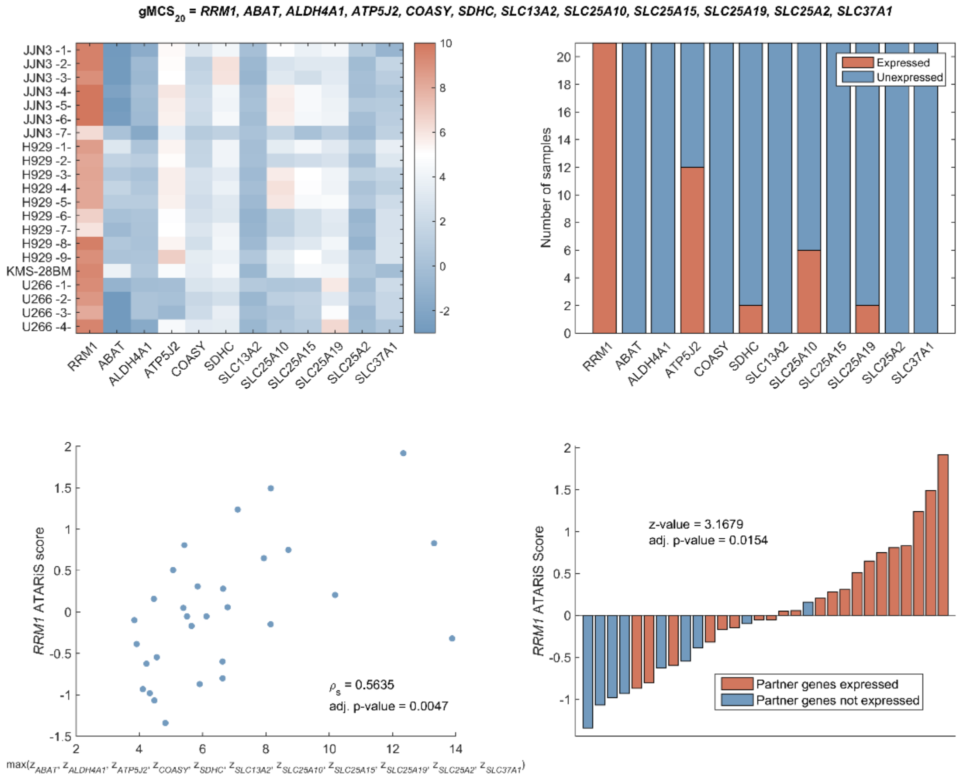

Supplementary Figure 22: gMCS$_{20}$ analysis. (a) Heatmap of Barcode z-scores[5] of *RRM1* and its partner genes (involved in gMCS$_{20}$) in different MM samples analyzed; **(b)** Number of MM samples where *RRM1* and its partner genes (involved in gMCS$_{20}$) are expressed/unexpressed, according to Barcode threshold of expression ($z \geq 5$). This gMCS explains the essentiality of *RRM1* in 6 samples; **(c)** Spearman´s correlation analyzing the dependence of *RRM1* ATARiS score on gene expression levels of partner genes in gMCS$_{20}$; **(d)** Bar plot of *RRM1* ATARiS score when partner genes are (or not) expressed in gMCS$_{20}$. To decide whether a gene is expressed, we used the standard threshold provided by the Gene Expression Barcode algorithm 3.0.

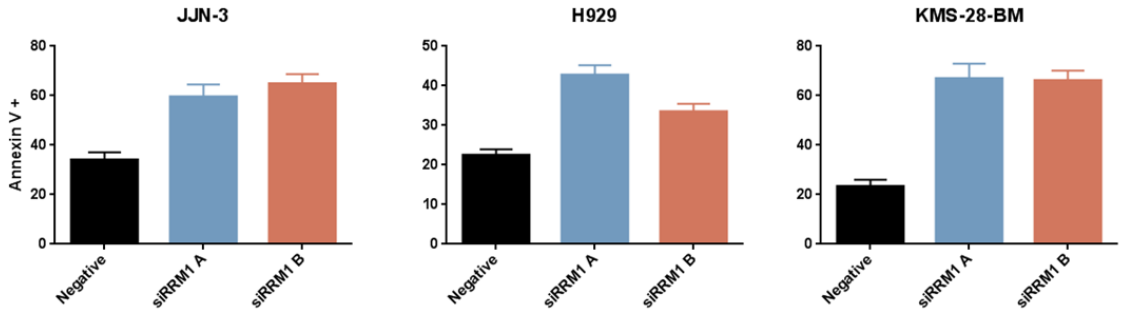

Supplementary Figure 23: Apoptosis analysis of JJN-3, H929 and KMS-28-BM cell lines nucleofected with siRNAs targeted to *RRM1* gene. Data represent mean ± standard deviation of at least three experiments.

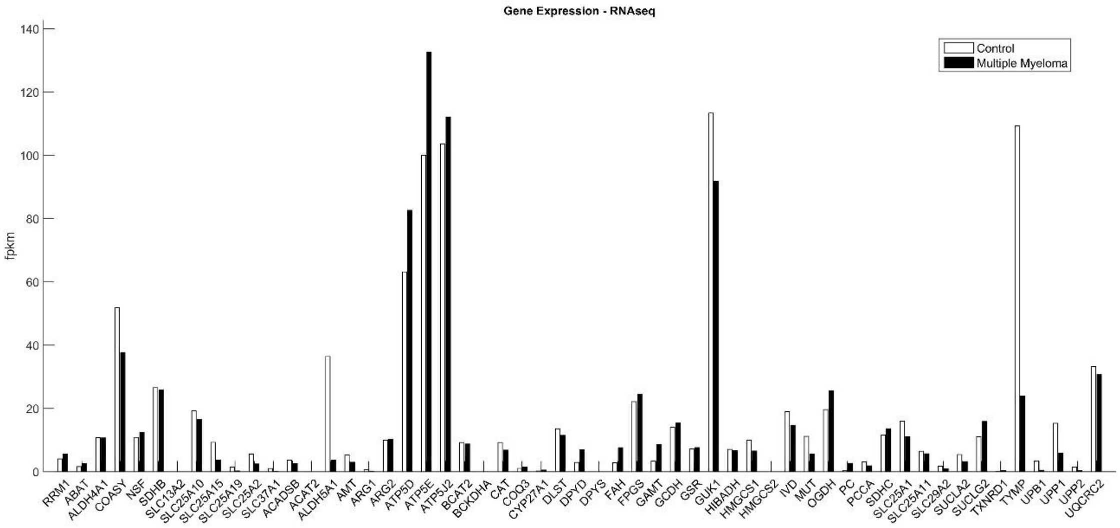

Supplementary Figure 24: Average RNAseq expression levels (fpkm) of all genes included in the gMCSs calculated from 11 MM samples and 4 samples of normal plasma cells[13]. The first 12 genes are part of the gMCS shown in the main paper.

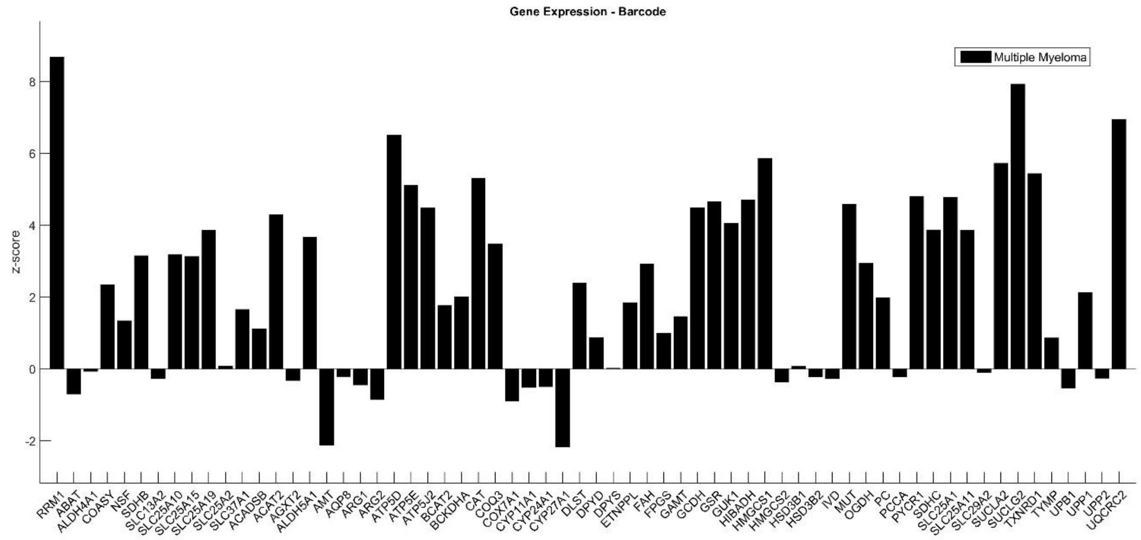

Supplementary Figure 25: Average Barcode expression levels (z-scores[5]) of all genes included in the gMCSs calculated from the 21 samples of MM cell lines analyzed in this work. The first 12 genes are part of the gMCS shown in the main paper.

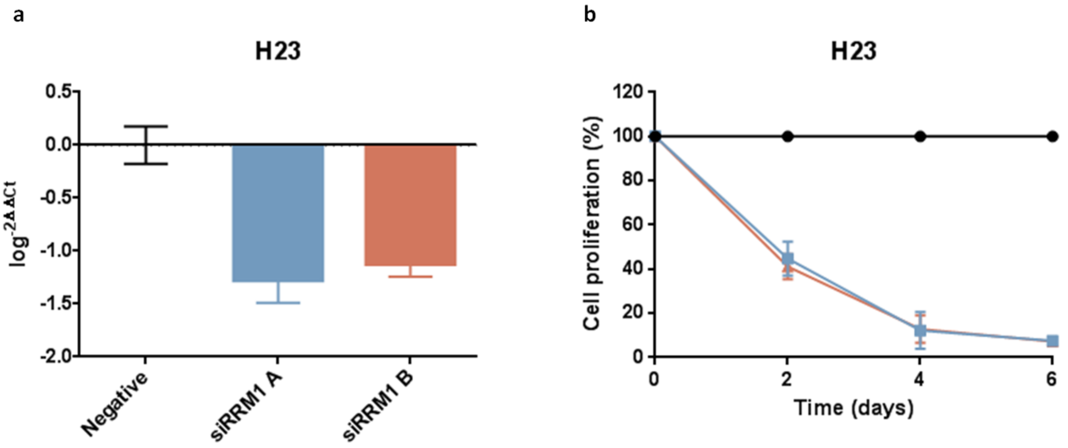

Supplementary Figure 26: Gene silencing analysis of *RRM1* in H23 cell line. (a) mRNA expression of *RRM1* gene 48h after transfection with the specific siRNAs. Data are referred to *GUS* gene and an experimental group nucleofected with negative control siRNA. **(b)** Proliferation of H23 cell line transfected with siRNAs targeted to *RRM1* gene was studied by MTS. The proliferation percentage refers to cells transfected with a negative control siRNA. Data represent mean ± standard deviation of at least three experiments.

Supplementary Table 1. List of the top 30 cell lines included in Project Achilles v2.4.3 and Cancer Cell Line Encyclopedia.

Achilles Accession	Cell Line	GSM
EFO21_OVARY	EFO-21	GSM887000
EFE184_ENDOMETRIUM	EFE-184	GSM886997
NCIH23_LUNG	NCI-H23	GSM887421
RKO_LARGE_INTESTINE	RKO	GSM887541
HT29_LARGE_INTESTINE	HT-29	GSM887141
LAMA84_HAEMATOPOIETIC_AND_LYMPHOID_TISSUE	LAMA-84	GSM887262
SKCO1_LARGE_INTESTINE	SK-CO-1	GSM887576
HCC2218_BREAST	HCC2218	GSM887049
PANC0813_PANCREAS	Panc 08.13	GSM887499
ZR7530_BREAST	ZR-75-30	GSM887751
HCC1954_BREAST	HCC1954	GSM887046
HT55_LARGE_INTESTINE	HT55	GSM887142
TE10_OESOPHAGUS	TE10	GSM887691
HCC70_BREAST	HCC70	GSM887058
NCIH1299_LUNG	NCI-H1299	GSM887355
COV362_OVARY	COV362	GSM886963
AGS_STOMACH	AGS	GSM886864
MDAMB453_BREAST	MDA-MB-453	GSM887300
MCF7_BREAST	MCF7	GSM887291
MONOMAC6_HAEMATOPOIETIC_AND_LYMPHOID_TISSUE	MONO-MAC-6	GSM887338
GP2D_LARGE_INTESTINE	GP2d	GSM887027
NCIH661_LUNG	NCI-H661	GSM887441
EFM19_BREAST	EFM-19	GSM886999
SNU840_OVARY	SNU-840	GSM887640
JHOC5_OVARY	JHOC-5	GSM887175
BT474_BREAST	BT-474	GSM886892
NCIH1437_LUNG	NCI-H1437	GSM887364
PANC0327_PANCREAS	Panc 03.27	GSM887496
BT20_BREAST	BT-20	GSM886891
MIAPACA2_PANCREAS	MIA PaCa-2	GSM887320

Supplementary Table 2. Contingency table with the essentiality predictions of GIMME in the Project Achilles data.

		GIMME	
		**Essential**	**Non-Essential**
**Achilles**	**Essential**	49	551
	**Non-Essential**	24	576

Supplementary Table 3. Contingency table with the essentiality predictions of iMAT in the Project Achilles data.

		iMAT	
		**Essential**	**Non-Essential**
**Achilles**	**Essential**	35	565
	**Non-Essential**	29	571

Supplementary Table 4. List of GSMs involved in the *RRM1* analysis in Multiple Myeloma.

Accession	Cell Line	GSM
JJN3 -1-	JJN-3	GSM229051
JJN3 -2-	JJN-3	GSM915718
JJN3 -3-	JJN-3	GSM915719
JJN3 -4-	JJN-3	GSM915720
JJN3 -5-	JJN-3	GSM1094684
JJN3 -6-	JJN-3	GSM1094685
JJN3 -7-	JJN-3	GSM1374579
H929 -1-	H929	GSM351746
H929 -2-	H929	GSM451261
H929 -3-	H929	GSM451264
H929 -4-	H929	GSM451267
H929 -5-	H929	GSM511161
H929 -6-	H929	GSM511162
H929 -7-	H929	GSM511163
H929 -8-	H929	GSM562817
H929 -9-	H929	GSM662887
KMS-28BM	KMS-28-BM	GSM887227
U266 -1-	U266	GSM363377
U266 -2-	U266	GSM363399
U266 -3-	U266	GSM562821
U266 -4-	U266	GSM887721

Supplementary Table 5. Summary of adjusted p-values for each gMCS found in the *RRM1* essentiality study in Multiple Myeloma (MM) for different statistical analyses conducted in the main text.

	Binomial Test		Achilles Scatter Plot		Achilles Bar Plot	
	**p-value**	**adj. p-value**	**p-value**	**adj. p-value**	**p-value**	**adj. p-value**
**$gMCS_1$**	0.9255	0.9810	0.7422	0.7624	0.7892	0.9285
**$gMCS_2$**	0.00001	0.0007	0.7624	0.7624	0.3587	0.4782
**$gMCS_3$**	0.00001	0.0007	0.7009	0.7624	0.3587	0.4782
**$gMCS_4$**	0.00001	0.0007	0.7009	0.7624	0.3587	0.4782
**$gMCS_5$**	0.4334	0.9631	0.0012	0.006	0.0062	0.031
**$gMCS_6$**	0.0004	0.0019	0.0018	0.0071	0.003	0.0298
**$gMCS_7$**	0.0206	0.0589	0.0953	0.1466	0.0922	0.2049
**$gMCS_8$**	0.0561	0.1404	0.0037	0.0122	0.0207	0.0694
**$gMCS_9$**	0.0064	0.0214	0.064	0.1067	0.1542	0.3085
**$gMCS_{10}$**	0.9255	0.9810	0.0004	0.0047	0.0208	0.0694
**$gMCS_{11}$**	0.0064	0.0214	0.1501	0.2001	0.3848	0.4810
**$gMCS_{12}$**	0.9810	0.9810	0.0318	0.0794	-*	-*
**$gMCS_{13}$**	0.9810	0.9810	0.0318	0.0794	-*	-*
**$gMCS_{14}$**	0.8083	0.9810	0.1919	0.2398	0.0375	0.1071
**$gMCS_{15}$**	0.9255	0.9810	0.0534	0.097	0.2521	0.4202
**$gMCS_{16}$**	0.9255	0.9810	0.117	0.1671	0.0529	0.1322
**$gMCS_{17}$**	0.9255	0.9810	0.0534	0.097	0.2521	0.4202
**$gMCS_{18}$**	0.9255	0.9810	0.0006	0.0047	0.0056	0.031
**$gMCS_{19}$**	0.9255	0.9810	0.0472	0.097	-*	-*
**$gMCS_{20}$**	0.6326	0.9810	0.0007	0.0047	0.0008	0.0154

* In these cases, all cell lines were assigned to a single class and, therefore, Mann-Whitney test could not be calculated.

Supplementary Table 6. Prediction of the essentiality of *RRM1* at the sample and cell line level in the MM study. Green coloring implies essentiality of *RRM1*, while red coloring non-essentiality.

GSM	gMCS	GIMME	iMAT	Cell Line	gMCS	GIMME	iMAT
GSM229051	Green	Green	Green	JJN3	Green	Green	Green
GSM915718	Green	Green	Red				
GSM915719	Green	Green	Red				
GSM915720	Green	Green	Red				
GSM1094684	Green	Red	Red				
GSM1094685	Green	Red	Red				
GSM1374579	Green	Red	Red				
GSM351746	Green	Red	Red	H929	Green	Red	Green
GSM451261	Green	Red	Red				
GSM451264	Green	Red	Red				
GSM451267	Green	Red	Green				
GSM511161	Green	Green	Green				
GSM511162	Green	Green	Red				
GSM511163	Green	Green	Green				
GSM562817	Green	Green	Red				
GSM662887	Green	Red	Red				
GSM887227	Green	Green	Red	KMS-28BM	Green	Green	Red
GSM363377	Red	Green	Green	U266	Red	Green	Red
GSM363399	Green	Green	Red				
GSM562821	Green	Green	Red				
GSM887721	Red	Green	Red				

Supplementary Table 7. Summary of adjusted Spearman's correlation p-values in Achilles Scatter plots of different gMCSs using max, mean and sum of the expression of the partner genes of *RRM1*.

	Achilles Scatter Plot - MAX		Achilles Scatter Plot - MEAN		Achilles Scatter Plot - SUM	
	p-value	adj. p-value	p-value	adj. p-value	p-value	adj. p-value
$gMCS_1$	0.7422	0.7624	0.7422	0.87315	0.7422	0.87315
$gMCS_2$	0.7624	0.7624	0.8914	0.89136	0.8914	0.89136
$gMCS_3$	0.7009	0.7624	0.8414	0.88566	0.8414	0.88566
$gMCS_4$	0.7009	0.7624	0.7894	0.8771	0.7894	0.8771
$gMCS_5$	0.0012	0.006	0.0012	0.00496	0.0012	0.00496
$gMCS_6$	0.0018	0.0071	0.004	0.01128	0.004	0.01128
$gMCS_7$	0.0953	0.1466	0.0151	0.03357	0.0151	0.03357
$gMCS_8$	0.0037	0.0122	0.0069	0.01727	0.0069	0.01727
$gMCS_9$	0.064	0.1067	0.1218	0.16238	0.1218	0.16238
$gMCS_{10}$	0.0004	0.0047	0.0006	0.004	0.0006	0.004
$gMCS_{11}$	0.1501	0.2001	0.1146	0.16238	0.1146	0.16238
$gMCS_{12}$	0.0318	0.0794	0.0039	0.01128	0.0039	0.01128
$gMCS_{13}$	0.0318	0.0794	0.1627	0.20338	0.1627	0.20338
$gMCS_{14}$	0.1919	0.2398	0.0361	0.0602	0.0361	0.0602
$gMCS_{15}$	0.0534	0.097	0.0210	0.0382	0.0210	0.0382
$gMCS_{16}$	0.117	0.1671	0.0205	0.0382	0.0205	0.0382
$gMCS_{17}$	0.0534	0.097	0.046	0.0707	0.046	0.0707
$gMCS_{18}$	0.0006	0.0047	0.0008	0.004	0.0008	0.004
$gMCS_{19}$	0.0472	0.097	0.0008	0.004	0.0008	0.004
$gMCS_{20}$	0.0007	0.0047	0.0004	0.004	0.0004	0.004

Supplementary References

1. Klamt, S. & Gilles, E. D. Minimal cut sets in biochemical reaction networks. *Bioinformatics (Oxford, England)* **20**, 226–234 (2004).
2. Tobalina, L., Pey, J. & Planes, F. J. Direct calculation of minimal cut sets involving a specific reaction knock-out. *Bioinformatics (Oxford, England)* **32**, 2001–2007 (2016).
3. Shlomi, T., Cabili, M. N., Herrgård, M. J., Palsson, B. O. & Ruppin, E. Network-based prediction of human tissue-specific metabolism. *Nat Biotech* **26**, 1003–1010 (2008).
4. Becker, S. A. & Palsson, B. O. Context-Specific Metabolic Networks Are Consistent with Experiments. *PLOS Computational Biology* **4**, e1000082– (2008).
5. McCall, M. N. *et al.* The Gene Expression Barcode 3.0: improved data processing and mining tools. *Nucleic acids research* **42**, 938–43 (2014).
6. Kamp, A. von & Klamt, S. Enumeration of Smallest Intervention Strategies in Genome-Scale Metabolic Networks. *PLOS Computational Biology* **10**, e1003378– (2014).
7. Thiele, I. *et al.* A community-driven global reconstruction of human metabolism. *Nat Biotech* **31**, 419–425 (2013).
8. Aye, Y., Li, M., Long, M. J. C. & Weiss, R. S. Ribonucleotide reductase and cancer: biological mechanisms and targeted therapies. *Oncogene* **34**, 2011–2021 (2015).
9. Nordlund, Pã¤. & Reichard, P. Ribonucleotide Reductases. *Annu. Rev. Biochem.* **75**, 681–706 (2006).
10. Kanehisa, M., Sato, Y., Kawashima, M., Furumichi, M. & Tanabe, M. KEGG as a reference resource for gene and protein annotation. *Nucleic acids research* **44**, 457–62 (2016).
11. Kohnken, R., Kodigepalli, K. M. & Wu, L. Regulation of deoxynucleotide metabolism in cancer: novel mechanisms and therapeutic implications. *Molecular cancer* **14**, 176– (2015).
12. Tiedemann, R. E. *et al.* Identification of Molecular Vulnerabilities in Human Multiple Myeloma Cells by RNA Interference Lethality Screening of the Druggable Genome. *Cancer Res* **72**, 757– (2012).
13. Agirre, X. *et al.* Whole-epigenome analysis in multiple myeloma reveals DNA hypermethylation of B cell-specific enhancers. *Genome Research* **25**, 478–487 (2015).
